# Elastic Properties and Electronic Properties of M*_x_*N*_y_* (M = Ti, Zr) from First Principles Calculations

**DOI:** 10.3390/ma11091640

**Published:** 2018-09-07

**Authors:** Yangqi Ji, Xiaoli Yuan

**Affiliations:** College of Science, Hohai University, Nanjing 210098, China; jiyangqi123@126.com

**Keywords:** M*_x_*N*_y_* (M = Ti; Zr), elastic properties, electronic properties, first principles calculations

## Abstract

The elastic properties and electronic properties of M*_x_*N*_y_* (M = Ti, Zr) TiN, Ti_2_N, Zr_3_N_4_, ZrN with different structures have been investigated using density functional theory. Through the calculation of the elastic constants, it was found that all of these structures meet the mechanical stability except for ZrN with space group P6_3_mc. Their mechanical properties are studied by a comparison of various parameters. The stiffness of TiN is larger than that of ZrN with space group Fm$\bar{3}$m. Ti_2_N’s stiffness with space group I4_1_/amdz is larger than Ti_2_N with space group P4_2_/mnm. Zr_3_N_4_’s stiffness with space group Pnam is largest in three structures of Zr_3_N_4_. TiN, Ti_2_N and ZrN are non-central force, Zr_3_N_4_ is central force. TiN and ZrN with space group Fm$\bar{3}$m are brittle, and TiN is brittler than ZrN with space group Fm$\bar{3}$m. The two kinds of Ti_2_N are brittle and Ti_2_N with space group I4_1_/amdz is larger. Three structures of Zr_3_N_4_ are tough and Zr_3_N_4 _with space group I$\bar{4}$3d is the toughest. Meanwhile, the electronic properties of TiN, Ti_2_N, Zr_3_N_4_ and ZrN were calculated, possible superconducting properties of the studied materials were predicted.

## 1. Introduction

In modern society and significant scientific research realms, superhard materials have been investigated by many people [1,2,3,4,5]. Titanium nitride has been widely used as a superhard material in the aerospace industry [6], oil-producing industry [7] and other fields due to its high strength, good heat-resistance and excellent corrosion resistance. Zirconium nitride has aroused interest because it manifests immense potentialities in cutting tools [8] and it is a good superconductor [9] owing to its high superconducting critical temperature. So, it is of high research value to investigate the properties of titanium nitride and zirconium nitride. Studying their properties can help us make better use of them as well as giving full play to their value.

Therefore, on account of the importance in fundamental science and technological applications, there has been plenty of research about the structural and physical properties of these two kinds of materials in the past few years. Saha et al. used first-principles calculations based on density functional theory to study ZrN’s electronic structure, vibrational spectra and thermal properties [10]. Guo et al. researched cubic zirconium nitride (c-Zr_3_N_4_) with the same structure as Th_3_P_4_. They found the structural and electronic properties by the local density functional pseudopotential method [11]. Wang et al. adopted an efficacious strain-stress way to compute the elastic stiffness constants of TiN, and got some related properties by first-principles calculations within the generalized gradient approximation [12]. Ivashchenko et al. studied the electromagnetic properties of Ti_2_N with two different structures under pressure [13]. Kim et al. obtained the color of TiN and ZrN by first-principles calculations [14]. Some others also have studied TiN, Ti_2_N, ZrN or Zr_3_N_4_’s properties in a specific aspect [15,16,17,18,19,20]. When we use the Inorganic Crystal Structure Database, we can find that these crystals have many different lattice parameters and space groups. The elastic properties and electronic structures of these substances have never been compared by unified research so far. So, we studied them and made comparative analyses.

The first-principles calculation with the pseudopotential method based on density functional theory (DFT) has been rapidly developed into a standard tool for material modeling simulation in the fields of physics, mechanics and material science [21,22,23]. Using density functional theory (DFT) and generalized gradient approximation (GGA) can calculate the various properties of the crystals. In this paper, we used the first-principles calculation with pseudopotential density functional theory (PDFT), the generalized gradient approximation (GGA) and the quasi-harmonic Debye model to build different structures of ZrN, Zr_3_N_4_, TiN and Ti_2_N. After that, the stabilities of these structures were predicted by simulation. By studying the elastic properties and electronic properties of ZrN, Zr_3_N_4_, TiN and Ti_2_N, the change rules were analyzed and summarized.

## 2. Computational Method

**Figure 1:**
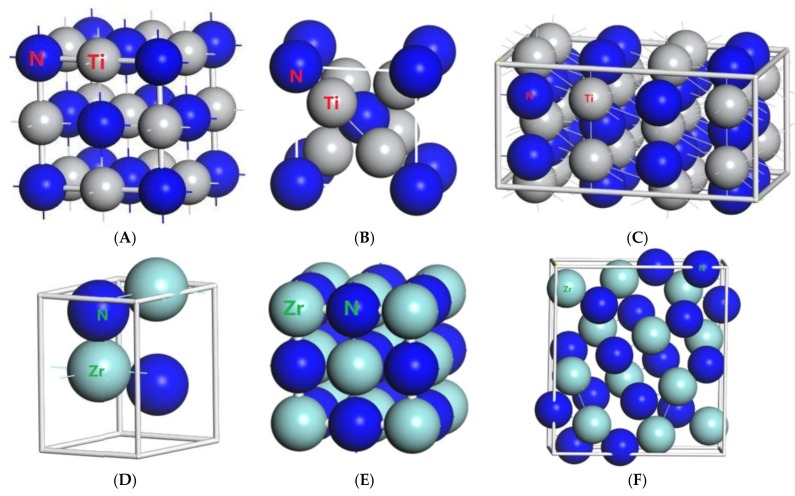

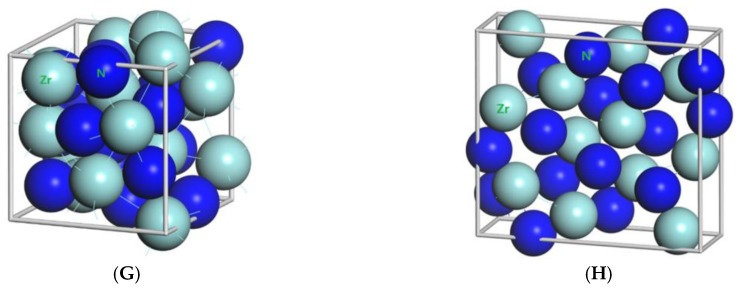
Primitive unit cells of (**A**) TiN with space group of Fm$\bar{3}$m; (**B**) Ti_2_N with space group of P4_2_/mnm; (**C**) Ti_2_N with space group of I4_1_/amdz; (**D**) ZrN with space group of P6_3_mc; (**E**) ZrN with space group of Fm$\bar{3}$m; (**F**) Zr_3_N_4_ with space group of Pna2_1_; (**G**) Zr_3_N_4_ with space group of I$\bar{4}$3d; (**H**) Zr_3_N_4_ with space group of Pnam.

With the purpose of calculating the exact properties of the materials, all calculations were computed by using the CASTEP [24] software package of Materials Studio software created by Accelrys (San Diego, CA, USA) according to the density functional theory (DFT). The atoms calculated are Zr (4s^2^4p^6^4d^2^5s^2^), Ti (3s^2^3p^6^3d^2^4s^2^) and N (2s^2^2p^3^). We used the combination of the Perdew-Burke-Ernzerhof (PBE) system and the generalized gradient function (GGA) to calculate the exchange-correlation energy [25]. Two kinds of Ti_2_N, two kinds of ZrN and three kinds of Zr_3_N_4_ were studied as shown in Figure 1. At the same time, Ti and Zr are subgroup elements and TiN’s space group is the same as ZrN, so TiN was also studied as a contrast. We set the suitable cutoff energy and K point as shown in Table 1. Based on these parameters, we can ensure that the total energy of these substances converges from a proper set. Calculated structural data under zero pressure are given in Table 2. We have studied the elastic properties using the optimized stable structure.

## 3. Results and Discussion

### 3.1. Result of the Elastic Properties

TiN, ZrN with space group Fm$\bar{3}$m and Zr_3_N_4_ with space group I$\bar{4}$3d belong to the cubic system, and their elastic tensors $C_{\mathrm{ij}}$ have three independent components $C_{11}$, $C_{12}$ and $C_{44}$. Its equations are derived from reference [26]. All Ti_2_N and ZrN with space group P6_3_mc belong to the tetragonal system, whose elastic tensors $C_{\mathrm{ij}}$ have six independent components $C_{11}$, $C_{12}$, $C_{13}$, $C_{33}$, $C_{44}$ and $C_{66}$. Zr_3_N_4_ with space group Pna2_1_ and Zr_3_N_4_ with space group Pnam belongs to the orthonormal system, whose elastic tensors $C_{\mathrm{ij}}$ have nine independent components $C_{11}$, $C_{12}$, $C_{13}$, $C_{22}$, $C_{23}$, $C_{33}$, $C_{44}$, $C_{55}$ and $C_{66}$. Its equations are derived from reference [27]. On account of the fact that the Voigt band is acquired by the average polycrystalline moduli, it is the upper band of the real modulus. Meanwhile, the Reuss band is acquired by hypothetical stress, so it describes the lower limit. The arithmetic mean of the two bands is called the Voigt-Reuss-Hill approximation. *B* represents bulk modulus, and *G* expresses shear modulus. By using the subscript which indicates the Voigt band, Reuss band and Hill average by V, R and H, respectively, they are calculated by the following equations.

For cubic system:

(1) $$B_{V}=B_{R}=\frac{C_{11}+2C_{12}}{3},$$

(2) $$G_{V}=\frac{C_{11}-C_{12}+3C_{44}}{5}.$$

(3) $$G_{R}=\frac{5C_{44}(C_{11}-C_{12})}{4C_{44}+3(C_{11}-C_{12})}$$

The criterion of mechanical stability is

$$C_{11}>0,C_{44}>0,C_{11}>\left|C_{12}\right|,(C_{11}+2C_{12})>0$$

For the tetragonal system:

(4) $$M=C_{11}+C_{12}+2C_{33}-4C_{13},$$

(5) $$C^{2}=(C_{11}+C_{12})C_{33}-2{C_{13}}^{2},$$

(6) $$B_{V}=\frac{2(C_{11}+C_{12})+C_{33}+4C_{13}}{9},$$

(7) $$G_{V}=\frac{M+3C_{11}-3C_{12}+12C_{44}+6C_{66}}{30},$$

(8) $$B_{R}=\frac{C^{2}}{M},$$

(9) $$G_{R}=\frac{15}{\frac{18B_{V}}{C^{2}}+\frac{6}{C_{44}}+\frac{3}{C_{66}}+\frac{6}{C_{11}-C_{12}}}.$$

The criterion of mechanical stability is

$$\begin{array}{l} C_{11}>0,C_{33}>0,C_{44}>0,C_{66}>0,(C_{11}-C_{12})>0\\ (C_{11}+C_{33}-2C_{13})>0,\\ {}[2(C_{11}+C_{12})+C_{33}+4C_{13}]>0 \end{array}$$

For the orthonormal system

(10) $$B_{V}=\frac{1}{9}[C_{11}+C_{12}+C_{33}+2(C_{12}+C_{13}+C_{23})]$$

(11) $$G_{V}=\frac{C_{11}+C_{22}+C_{33}+3(C_{44}+C_{55}+C_{66})-(C_{12}+C_{13}+C_{23})}{15}$$

(12) $$\begin{array}{l} B_{R}=\Delta [C_{11}(C_{22}+C_{33}-2C_{23})+C_{22}(C_{33}-2C_{13})-2C_{33}C_{12}+C_{12}(2C_{23}-C_{12})\\ {+C_{13}(2C_{12}-C_{13})+C_{23}(2C_{13}-C_{23})]}^{-1} \end{array}$$

(13) $$\begin{array}{l} G_{R}=15\{4[C_{11}(C_{22}+C_{33}+C_{23})+C_{22}(C_{33}+C_{13})+C_{33}C_{12}-C_{12}(C_{23}+C_{12})\\ {-C_{13}(C_{12}+C_{13})-C_{23}(C_{13}+C_{23})]/\Delta +3(\frac{1}{C_{44}}+\frac{1}{C_{55}}+\frac{1}{C_{66}})\}}^{-1} \end{array}$$

(14) $$\Delta =C_{13}(C_{12}C_{23}-C_{13}C_{22})+C_{23}(C_{12}C_{13}-C_{23}C_{11})+C_{33}(C_{11}C_{22}-{C_{12}}^{2}).$$

The criterion of mechanical stability is

$$\begin{array}{l} C_{11}>0,C_{22}>0,C_{33}>0,C_{44}>0,C_{55}>0,C_{66}>0\\ C_{11}+C_{22}+C_{33}+2(C_{12}+C_{13}+C_{23})>0\\ C_{11}+C_{22}-2C_{12}>0,C_{11}+C_{33}-2C_{13}>0\\ C_{22}+C_{33}-2C_{23}>0 \end{array}$$

*B_V_*, *B_R_*, *G_V_*, *G_R_* of different substances were obtained by the calculation of the Equations (1)–(14). Under the Voigt-Reuss-Hill approximation, it can be found that the modulus of polycrystal is the mean value under the Voigt bound and Reuss bound,

(15) $$B=\frac{1}{2}(B_{V}+B_{R}),$$

(16) $$G=\frac{1}{2}(G_{V}+G_{R}),$$

Young’s modulus (*E*) and poisson’s ratio (ν) were obtained by these equations:

(17) $$E=\frac{9BG}{3B+G},$$

(18) $$\nu =\frac{3B-2G}{2(3B+G)}.$$

The associated Equations (15)–(18) can calculate the relevant physical quantities. From the relevant literature, we know that *G* indicates the anti-plastic deformability of materials and *B* indicates the resistance to fracture of materials [28]. Pugh proposed the estimation of the ductility of a given material by the ratio of *G*/*B* between the shear modulus *G* and the bulk modulus *B* [29]. According to his theory, the small *G*/*B* values represent the toughness of the corresponding materials, while the larger *G*/*B* values represent brittleness. *G*/*B* = 0.57 is the critical value. The results we got were written in Table 3, Table 4, Table 5 and Table 6.

First of all, according to the results, ZrN with space group P6_3_mc fails to meet the conditions of mechanical stability. So, it is necessary to compare ZrN and TiN in the same space group. The data show that the Young’s modulus of TiN is larger than that of ZrN. It indicates that the stiffness of TiN is larger than that of ZrN with space group Fm$\bar{3}$m. Poisson’s ratios can show the binding force of the atom [30]. Poisson’s ratios of TiN and ZrN are both less than 0.25, so they are non-central force. *G*/*B* of TiN is 0.665, which shows that TiN is brittle. With regard to ZrN, *G*/*B* is 0.615. Therefore, ZrN with space group Fm$\bar{3}$m is brittle, and there is a reduction from TiN to ZrN. For Ti_2_N, we found that Ti_2_N’s stiffness with space group I4_1_/amdz is larger than that with space group p4_2_/mnm. Poisson’s ratios are the same, and they are non-central force. *G*/*B* of Ti_2_Nwith space group I4_1_/amdz is 0.69. At the same time, *G*/*B* of Ti_2_Nwith space group P4_2_/mnm is 0.665. The two kinds of Ti_2_N are brittle and Ti_2_N with space group I4_1_/amdz is larger. As for Zr_3_N_4_, Zr_3_N_4_ with space group Pnam has the largest stiffness; the second largest is Zr_3_N_4_ with space group Pna2_1_. Poisson’s ratios are the opposite, but all of them are central force. Meanwhile, three structures of Zr_3_N_4_ are tough and Zr_3_N_4_ with space group I$\bar{4}$3d is the toughest. The remaining two substances are not very different, which proves that the symmetry of the structure has little effect on this property.

### 3.2. Result of the Electronic Properties

Due to explaining the macroscopic properties, we investigated the electronic band structures, density of states and difference of the charge density of all structures under zero pressure. Figure 4 shows the calculated band structures along the high symmetry directions in the Brillouin zone. It is worth mentioning that DFT cannot give reliable results for the energy gap because DFT does not consider the correlation effect of electrons in 3d orbit and 4f orbitin some strongly correlated systems. As a result, band gaps became smaller. So, the judgement on whether the system is metallic or insulating and on the value of the gap was semi-quantitative.

**Figure 2:**
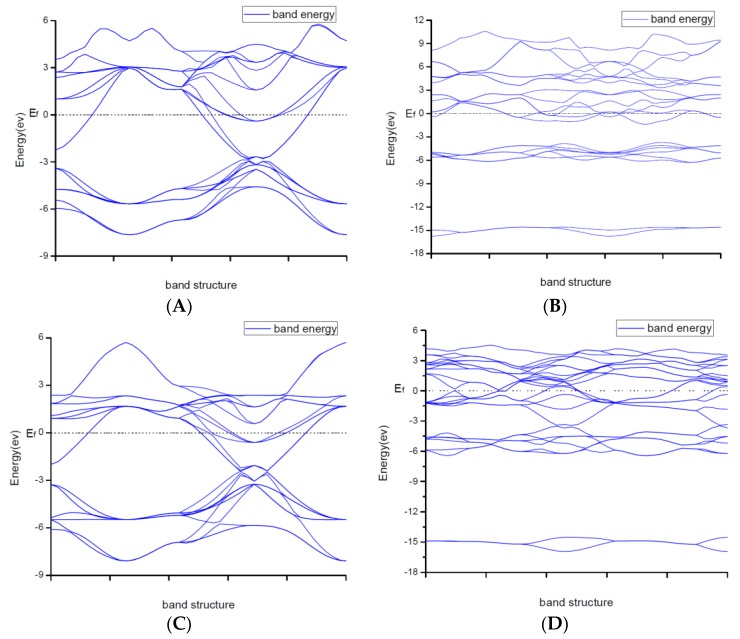

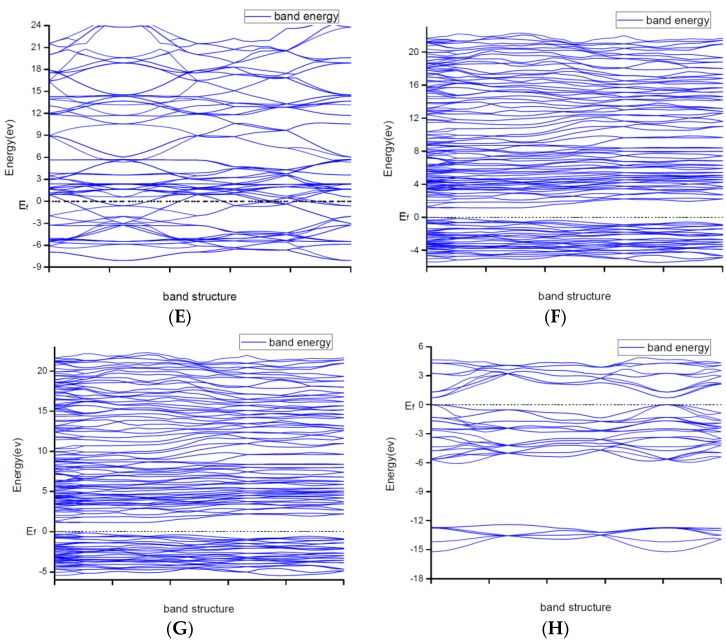
Band structures of (**A**) ZrN with space group Fm$\bar{3}$m; (**B**) ZrN with space group P6_3_mc; (**C**) TiN; (**D**) Ti_2_N with space group P4_2_/mnm; (**E**) Ti_2_N with space group I4_1_/amdz; (**F**) Zr_3_N_4_ with space group Pna2_1_; (**G**) Zr_3_N_4_ with space group Pnam; (**H**) Zr_3_N_4_ with space group I$\bar{4}$3d.

From Figure 2, the conduction band minimum and valence band maximum of TiN, ZrN with space group Fm$\bar{3}$m and Ti_2_N with two kinds of structures are located at the G-point with a very small band gap. The conduction band minimum and valence band maximum of ZrN with space group P6_3_mc is located at the M-point. The conduction band minimum and valence band maximum of Zr_3_N_4_ with space group I$\bar{4}$3d is located at the G-point. From the results, their band gaps are less than 1 eV. So, for these substances, electrons can easily gain energy at room temperature and jump to the transfer band to conduct electricity. The conduction band minimum and valence band maximum of Zr_3_N_4_ with space groups Pna2_1_ and Pnam are located at the Z-point with band gap between1eVand 3eV. These two substances are between conductors and insulators. Therefore, they are electrically conductive as long as the appropriate energy is given or the gaps between their energy are changed. It indicates that the electrical conductivity of ZrN with space group Fm$\bar{3}$m is better than that of ZrN with space group P6_3_mc. For TiN and ZrN with the same space group, TiNhas smaller electron effective mass and larger atomic non-localization than ZrN due to the bigger width of band structure. For three different structures of Zr_3_N_4_, symmetry and asymmetry have little influence on the electronic properties. The electrical conductivities of Zr_3_N_4_ with space groups Pna2_1_ and Pnam are better than that of Zr_3_N_4_ with space group I$\bar{4}$3d. Ti_2_N with space group I4_1_/amdz has a smaller electron effective mass and a degree of non-localization with stronger atomic orbital extension than Ti_2_N with space group P4_2_/mnm.

**Figure 3:**
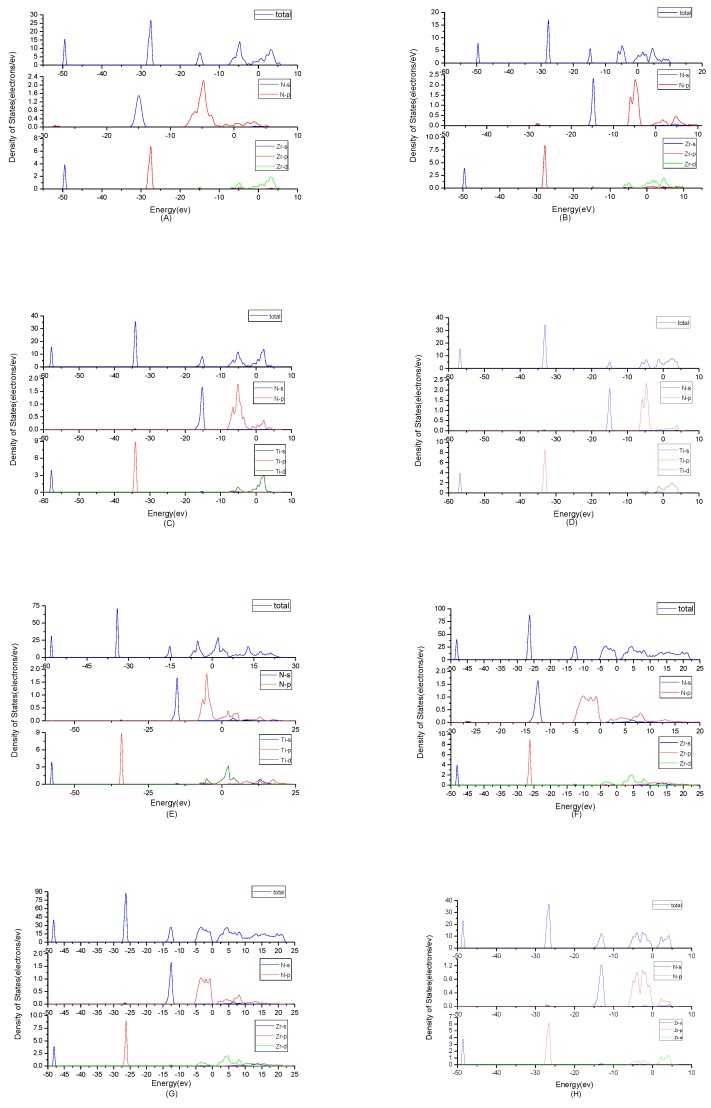
Total density of states plots and partial density of states plots of (**A**) ZrN with space group Fm$\bar{3}$m; (**B**) ZrN with space group P6_3_mc; (**C**) TiN, (**D**) Ti_2_N with space group P4_2_/mnm; (**E**) Ti_2_N with space group I4_1_/amdz; (**F**) Zr_3_N_4_ with space group Pna2_1_; (**G**) Zr_3_N_4_ with space group Pnam; (**H**) Zr_3_N_4_ with space group I$\bar{4}$3d.

To find origins of band structures, the total density of states and partial density of states were calculated as shown in Figure 3. PBE cannot accurately show the weak interaction between molecules, because it will make the values of d track and f track be slightly lower than the results of dispersion correction and there will besome differences in the hybridization at the top of the conduction band. But the corrected value is very small and will not affect the conclusion of this work. At the same time, the focus of this study is near the Fermi level, where the situation will not change substantially due to the effect of dispersion. By observing the density of states of ZrN, the results showed that the contributions of the atoms to the energy band are similar. N2p and Zr4d have obvious peaks at −5 eV, and they have a state density resonance, so N2p and Zr4d are bonded. Zr4d, Zr4P and Zr5s have a state density contribution to N2p, so they also form covalent bonds. Meanwhile, ZrN with space group P6_3_mc has a greater span of density of state and stronger domain. Accordingly, it has stronger bonds. For TiN, N2p have obvious peaks at Ti3d; they are bonded. In addition, Ti4p and Ti4s have a part in the contribution of state density to N2p. Ti’s s orbit and p orbit are the main sources of the valence band. N’s s orbits, p orbits and Ti’s have a small contribution. The d orbit of Ti and the p orbit of N are the main sources of the guide band. As for Ti_2_N, Ti_4_p, Ti4s and Ti3d are bonded respectively to N2s and N2p in the two structures. The s, p orbit of Ti (s_Ti_ and p_Ti_) and N (s_N_ and p_N_) are the main sources of the valence band. d_Ti_ orbit has a small contribution. For Ti_2_N with space group P4_2_/mnm, the d orbit of Ti (d_Ti_) and the p orbit of N (p_N_) are the main sources of the guide band. For another structure of Ti_2_N, the s_Ti_, d_Ti_ and p_Ti_ orbits are the main sources of the guide band. The s_N_ orbits and p_N_ orbits have some contributions. Finally, according to the density of states of Zr_3_N_4_, the differences between Zr_3_N_4_ with space group Pna2_1_ and Zr_3_N_4_ with space group Pnam are minimal; this situation shows that the symmetry of the space group has little effect on it. For Zr_3_N_4_ with space group I$\bar{4}$3d, s_N_ orbits, p_Zr_ orbits and d_Zr_ orbits form covalent bonds. The 2p_N_ forms covalent bonds with 5s_Zr_, 4p_Zr_and 4d_Zr_. For the other two structures, 2p_N_ forms covalent bonds with 5s_Zr_, 4d_Zr_ and 4p_Zr_ and 2s_N_ forms covalent bonds with 4d_Zr_ and 4p_Zr_. At the same time, Zr_3_N_4_ with space group Pna2_1_ and Zr_3_N_4_ with space group Pnam have a greater span of density of state and stronger domain. Therefore, they have stronger bonds.

**Figure 4:**
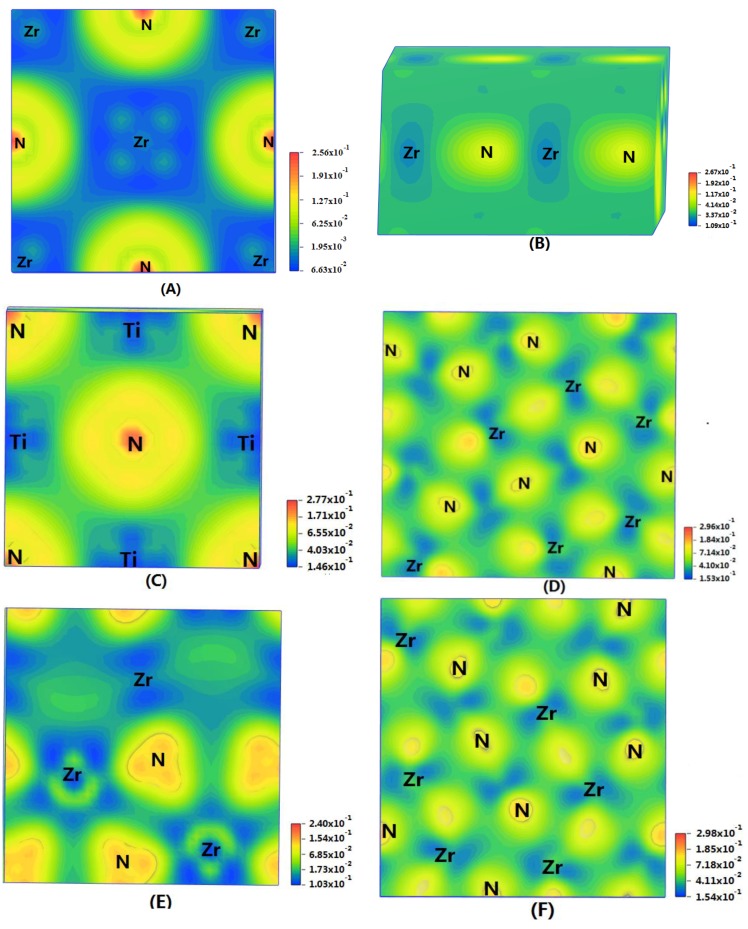

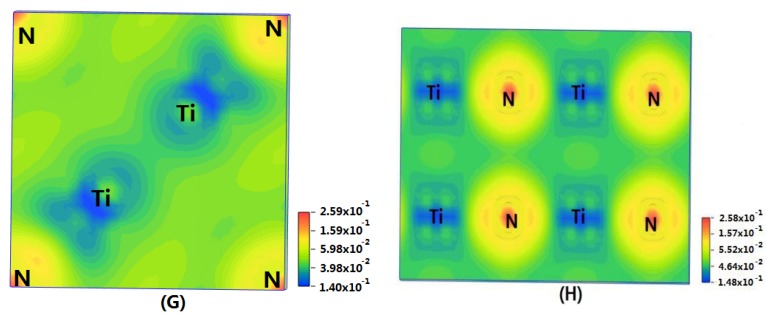
Difference charge density maps of (**A**) ZrN with space group Fm$\bar{3}$m; (**B**) ZrN with space group P6_3_mc; (**C**) TiN; (**D**) Zr_3_N_4_ with space group Pna2_1_; (**E**) Zr_3_N_4_ with space group I$\bar{4}$3d; (**F**) Zr_3_N_4_ with space group Pnam; (**G**)Ti_2_N with space group P4_2_/mnm; (**H**)Ti_2_N with space group I4_1_/amdz.

In order to show the bonding between atoms of different crystals more intuitively, difference charge density maps are shown in Figure 4. According to plot A and plot C, for TiN and ZrNwith space group Fm$\bar{3}$m, the charge densities between Ti and N are larger than that of Zr and N, which means that the effects between Ti–N are stronger. Meanwhile, ZrN with space group Fm$\bar{3}$m has stronger Zr–N bonds than ZrN with space group P6_3_mc. So, the interatomic interaction of ZrN with space group Fm$\bar{3}$m is greater than that of ZrN with space group P6_3_mc. For three different structures of Zr_3_N_4_, symmetry and asymmetry have little influence on the electronic properties. There are larger charge densities between Zr and N in Zr_3_N_4_ with space group Pna2_1_ and Zr_3_N_4_ with space group Pnam than the other structure. So, there are stronger interactions between their atoms. The last two graphs (Figure 4G,H) represent the charge density of TiN with two different structures. Ti_2_N with space group P4_2_/mnm has a larger charge density than Ti_2_N with space group I4_1_/amdz. Therefore, the interatomic effect of Ti_2_N with space group P4_2_/mnm is stronger.

### 3.3. Superconducting Properties

After obtaining the elastic properties and electronic properties of the studied materials, the possible superconducting properties were discussed based on the results. According to the simplified theory of superconductivity, if a material can become a superconductor, three conditions must be satisfied. First of all, atoms that make up crystals are lighter. Second, the coefficient of elasticity of the crystal is as large as possible, and crystals are relatively tough. Third, the effective Fermi level of materials should be low. Zr and Ti are two of the 28 superconducting elements, as their nitrogen compounds satisfy the first condition. From the results of the elastic properties, TiN is tougher than ZrN when used as superconducting material. Ti_2_N with space group I4_1_/amdz is better than Ti_2_N with space group P4_2_/mnm. The three structures of Zr_3_N_4_ all have potential as superconducting materials; Zr_3_N_4_ with space group I$\bar{4}$3d is the best of them. From the third point of view, metal systems are more likely to superconduct. So, TiN, ZrN and Zr_3_N_4_ with space group I$\bar{4}$3d are better choices to satisfy the third condition. Combining three characteristics, Zr_3_N_4_ with space group I$\bar{4}$3d is most likely to have superconductivity in all materials. TiN is more likely to be used as a superconducting material than ZrN with same space group. Ti_2_N with space group I4_1_/amdz is more suitable to be used as superconducting material than Ti_2_N with space group P4_2_/mnm.

## 4. Conclusions

In short, we have investigated the elastic properties and electronic properties of the four materials TiN, Ti_2_N, ZrN and Zr_3_N_4_ with different structures by using the first principles method.

In order to study the elastic properties, we have calculated elastic stiffness constants *C*_ij_, bulk modulus *B*, shear modulus *G*, Young modulus *E*, Poisson’s ratio ν and *G*/*B*. Research on Young’s modulus indicated that the stiffness of TiN is larger than that of ZrN with space group Fm$\bar{3}$m. Ti_2_N’s stiffness with space group I4_1_/amdz is larger than that with space group p4_2_/mnm. Zr_3_N_4_ with space group Pnam’s stiffness is largest; the second is Zr_3_N_4_ with space group Pna2_1_. For Poisson’s ratio, TiN, Ti_2_N and ZrN are non-central force, Zr_3_N_4_ are central force. Meanwhile, *G*/*B* shows that TiN and ZrN with space group Fm$\bar{3}$m are brittle, and TiN is larger than ZrN. The two kinds of Ti_2_N are brittle and Ti_2_N with space group I4_1_/amdz is larger. Three structures of Zr_3_N_4_ are tough and Zr_3_N_4_ with space group I$\bar{4}$3d is the toughest. It is worth mentioning that ZrN with space group P6_3_mc fails to meet the conditions of mechanical stability.

Based on the analysis of band structures, density of states and the difference in the charge density of these substances, their electronic properties are clear at a glance. The electrical conductivity of ZrN with space group Fm$\bar{3}$m is better than that of ZrN with space group P6_3_mc. The interatomic interaction of ZrN with space group Fm$\bar{3}$m is greater than that of ZrN with space group P6_3_mc. Contributions of the atom of ZrN to the energy band are similar. However, ZrN with space group P6_3_mc has a greater span of density of state and stronger domain. Accordingly, it has stronger bonds. For TiN and ZrN with the same space group, TiN has a smaller electron effective mass and larger atomic non-localization than ZrN due to the bigger width of band structure. The charge density between Ti and N is larger than that of Zr and N, which means that the effect between Ti–N is stronger. For three different structures of Zr_3_N_4_, symmetry and asymmetry have little influence on the electronic properties. Because the electrical conductivities of Zr_3_N_4_ with space groups Pna2_1_ and Pnam are better than that of Zr_3_N_4_ with space group I$\bar{4}$3d, the interactions between their atoms are stronger. They have a greater span of density of state and stronger domain. Therefore, they have stronger bonds. Ti_2_N with space group I4_1_/amdz has smaller electron effective mass and the degree of non-localization with stronger atomic orbital extension than Ti_2_N with space group P4_2_/mnm. The interatomic effect of Ti_2_N with space group P4_2_/mnm is stronger. For Ti_2_N with space group P4_2_/mnm, the d orbit of Ti and the p orbit of N are the main sources of the guide band. For another structure of Ti_2_N, the s, d and p orbit of Ti are the main sources of the guide band. N’s s orbits and p orbits have some contribution. They have different orbital bonding methods.

From the elastic and electronic results, Zr_3_N_4_ with space group I$\bar{4}$3d is most likely to have superconductivity in all materials. TiN is more likely to be used as superconducting material than ZrN with same space group. Ti_2_N with space group I4_1_/amdz is more suitable to be used as superconducting material than Ti_2_N with space group P4_2_/mnm.

## Figures and Tables

**Table 1:** Lattice parameters *a*, *b*, *c*, cell volume (*V*), the cutoff energy (eV) and K point of Ti_2_N, ZrN and Zr_3_N_4_.

Compound	Space Group	*a *(Å)	*b *(Å)	*c *(Å)	*V* (Å^3^)	Cutoff Energy (eV)	K Point
TiN	Fm$\bar{3}$m	4.25	-	-	76.77	750	20 × 20 × 20
Ti_2_N	I4_1_/amz	4.149	4.149	8.786	74.20	600	8 × 8 × 13
	P4_2_/mm	4.945	4.945	3.034	151.26	680	9 × 9 × 4
ZrN	Fm$\bar{3}$m	4.573	-	-	95.76	800	20 × 20 × 20
	P6_3_mc	3.128	3.128	5.013	42.49	720	22 × 22 × 6
Zr_3_N_4_	I$\bar{4}$3d	6.74	-	-	306.18	500	15 × 15 × 15
	Pna2_1_	9.729	10.818	3.281	345.32	480	15 × 15 × 15
	Pnam	9.788	10.854	3.300	350.59	490	6 × 17 × 5

**Table 2:** Data calculated in this work (some experimental data and some the results of others) of TiN, Ti_2_N, ZrN and Zr_3_N_4_ under zero pressure. (TW = this work, Exp = exsperiment, Cal. = other calculated, Space group = S.G).

Compound	Data Type	S.G	*a* (Å)	*b* (Å)	*c* (Å)
TiN	TW	Fm$\bar{3}$m	4.246	-	-
	Exp.	Fm$\bar{3}$m	4.250	-	-
	Cal.	Fm$\bar{3}$m	4.256 [12]	-	-
Ti_2_N	TW	I4_1_/amdz	6.003	6.003	8.502
	TW	P4_2_/mnm	4.952	4.952	3.034
	Cal.	P4_2_/mnm	4.928 [13]	4.928 [13]	3.021 [13]
ZrN	TW	Fm$\bar{3}$m	4.591	-	-
	Cal.	Fm$\bar{3}$m	4.59	-	-
	TW	P6_3_mc	3.564 [10]	3.564 [10]	5.538 [10]
Zr_3_N_4_	TW	I$\bar{4}$3d	6.783	-	-
		Pna2_1_	9.823	10.843	3.291
		Pnam	9.814	10.840	3.294
	Exp.	I$\bar{4}$3d	6.740 [11]	-	-

**Table 3:** Elastic stiffness constants *C*_ij_ (GPa) of ZrN with space group Fm$\bar{3}$m and TiN.

Compound	S.G	*C*_11_	*C*_12_	*C*_44_
TiN	Fm$\bar{3}$m	563.93	133.28	165.91
ZrN	Fm$\bar{3}$m	536.17	105.19	121.21

**Table 4:** Elastic stiffness constants *C*_ij_ (GPa) of Ti_2_N and ZrN with space group P6_3_mc.

Compound	S.G	*C*_11_	*C*_12_	*C*_13_	*C*_33_	*C*_44_	*C*_66_
Ti_2_N	I4_1_/amdz	512.26	188.64	120.05	568.49	166.22	231.47
	P4_2_/mnm	341.57	147.80	103.12	429.89	141.11	146.57
ZrN	P6_3_mc	219.22	162.29	165.69	120.14	51.24	28.46

**Table 5:** Elastic stiffness constants *C*_ij_ (GPa) of Zr_3_N_4_.

S.G	*C*_11_	*C*_12_	*C*_13_	*C*_22_	*C*_23_	*C*_33_	*C*_44_	*C*_55_	*C*_66_
I$\bar{4}$3d	332.4	119.7	-	-	-	-	64.91	-	-
Pna2_1_	211.4	126.1	125.0	383.9	116.2	425.5	56.44	95.7	132.8
Pnam	215.2	126.9	126.8	389.3	109.1	428.5	55.7	96.6	134.9

**Table 6:** Bulk modulus *B* (GPa), Shear modulus *G* (GPa), Young’s modulus *E* (GPa), Poisson’s ratio ν and *G*/*B* of ZrN, Zr_3_N_4_, TiN and Ti_2_N.

Compound	S.G	*B*	*G*	*E*	*ν*	*G*/*B*
ZrN	Fm$\bar{3}$m	248.85	152.92	380.77	0.245	0.615
	P6_3_mc	196.59	−569.37	-	-	-
TiN	Fm$\bar{3}$m	276.83	184.18	452.24	0.2277	0.665
Ti_2_N	I4_1_/amdz	272.27	187.96	458.40	0.220	0.690
	P4_2_/mnm	202.12	134.43	330.11	0.228	0.665
Zr_3_N_4_	I$\bar{4}$3d	190.6	79.18	208.7	0.318	0.415
	Pna2_1_	186.5	93.69	240.8	0.285	0.502
	Pnam	187.6	94.74	243.3	0.284	0.505
